# Numerical investigation of the optimal porosity of titanium foam for dental implants

**DOI:** 10.1016/j.heliyon.2024.e28063

**Published:** 2024-03-13

**Authors:** Hussein Farroukh, Fouad Kaddah, Toufic Wehbe

**Affiliations:** a Mechanical Engineering Department, Saint Joseph University of Beirut, Beirut, 17-5208, Lebanon; b Civil Engineering Department, Saint Joseph University of Beirut, Beirut, 17-5208, Lebanon

**Keywords:** Titanium foam, Porosity, Dental implant, Finite element analysis, Mechanical properties, AI (artificial intelligence)

## Abstract

**Background:**

This paper aims to indicate numerically the accurate porosity used for dental implants, following the emphasis on the preference for titanium foam on pure titanium implants. A 3D-optimized numerical model is created to demonstrate the detailed differences between models.

**Method:**

A 3D finite element model was generated using Abaqus for titanium and titanium foam implants with different porosities (50,60,62.5,70, and 80%) fixed in cortical and cancellous bone. The mechanical data for titanium foam is extracted from published literature. We evaluate an artificial intelligent equation for the stress-strain response of titanium foam with various porosities to describe their variations.

**Results:**

To evaluate the stress-strain variations for different porosities, exponential artificial intelligence provides high accuracy (>0.99). The numerical results show that titanium foam implants appear to transfer more loads to the bordering bones due to their lower stiffness and higher energy absorption, which can help reduce stress shielding problems. In surrounding bones, the maximum VM stress occurs at the neck region from 5.42 MPa for pure titanium to 21.53 MPa for titanium foam with 80% porosity. Additionally, a porosity of 62.5% appears to be the most suitable since Young's modulus for this porosity (13.82 GPa) is close to the cortical bone's modulus (14.5 GPa). This suitability is shown in FEA by the similarity in stress level between pure titanium and the corresponding porosity. Overall, titanium foam implants appear to be a promising option for improving the effectiveness and longevity of bone implants in surgical dentistry.

**Conclusion:**

Systematic numerical studies on titanium foam dental implants with different porosities. Analysis of the FE results shows that titanium foam with a porosity of 62.5% is more beneficial for use in dental implants.

## 1 Introduction

### 1.1 Background

Over the past few decades, implantology in dentistry has seen significant advancements, transforming treatment approaches for oral rehabilitation, and resolving issues with partial and complete edentulism. Titanium implants, which maintain natural teeth proportions, offer significant benefits over adhesive or mucous prostheses. The continuous connection between prosthetics, implant research, and industry has led to the incorporation of more diverse components for oral rehabilitation [1]. Surface topography affects cell shape, orientation, function, and proliferation. The chemical composition of the implant surface affects biocompatibility, and understanding a structure's surface properties requires both topographical and chemical techniques [1,2]. Surface treatments have been applied to titanium implants to adapt their characteristics and increase osteo-conductivity.

Studies show that implant surface qualities are crucial for optimal outcomes, with microscopical features affecting bone formation. Metal surface treatments aim to create physiologically active surfaces, with retention increasing in developing tissues. Smooth and rough implant surfaces are divided into additive and subtractive methods, hybrid procedures combining sandblasting and thermal etching, and acid etching and sanding. Smooth implants can be machined or electropolished, while plasma surface treatment (TPS) with titanium powders can present a risk of particle detachment [[2], [3], [4]].

Because of titanium's outstanding mechanical properties, low density, and high chemical resistance, titanium foams have a wide range of potential uses. These foams are primarily utilized in mechanical applications, such as sandwich materials in submarines or aircraft, and are widely used for this purpose [5]. This application utilizes titanium's excellent stiffness and strength relative to its density, along with its outstanding corrosion resistance [6]. Porous dental implants represent another area of application where titanium's exceptional biomechanical properties, especially its resistance to fatigue, are vital [7,8]. The reduced stiffness of titanium foams represents an advantage for reducing stress-shielding in the bordering bone. Indeed, stress-shielding can lead to bone resorption and loosening of the implant strength [[9], [10], [11]].

Dental implants can replace a natural tooth's root to securely anchor dental prostheses. Osseointegration fosters a strong bone-implant connection by directly linking living bone and implant surfaces, ensuring a durable interlock over time [12,13]. Osseointegration is influenced by bone health, implant design, loadings, and other biological factors [14]. The biological integration between the implant and tissue results from the porous features improved ability to transmit stress between the implant and the bone. Because of their larger surface, they also prevent premature mobility and micromotion and offer early mechanical stability. Thus, by providing a larger fixing surface, osseointegration is promoted. Additionally, a porous biomaterial facilitates vascularization and bone regeneration by drawing and allowing cells from the surrounding bone to penetrate its interior [2,4,14].

Fundamentally, implants should be made of biomaterials that are compatible with conditions in the human body. However, compared to mineralized tissue, these materials have a larger rigidity. A difference between the titanium implant's Young's modulus (110 GPa), natural cortical (13–18 GPa), and cancellous bone (around 2 GPa) might result from the typical non-porous titanium used for dental implants [[15], [16], [17], [18]].

Kayabasi et al. [19] demonstrates numerically that the high-stress values were located within the cortical bone at the neck of the implant. Piotrowski et al. [20] discovered that when a low Young's modulus implant was used, the stress at the boundary between the cortical bone and the implant decreased notably. This reduces micromotion within the cortical bone-implant interface. Shirazi et al. [21] showed that the FGBM (functionally graded biomaterial) dental implant can reduce the high-stress values and the stress shielding effect in natural bones. Salehi et al. [22] indicated analytically that the 50 and 60% porosity of samples of titanium foam were suitable for biomedical applications. To ensure the ingrowth of bone into the pores, Nouri [23] showed experimentally that the titanium foam must have at least 60% porosity. Huang et al. [7] found that implants with porous structures can greatly enhance osseointegration following 12 weeks of bone healing. Yaqoob et al. [24] mechanically approved that Ti foams can reduce the problem of stress shielding. Ouldyerou et al. [25] show that the 3D printing manufacturing of Ti foam reduces the stress shielding problem and that 77% porosity of Ti foam transfers more load to the nearby bone than 63%.

A variety of processes, such as foaming, additive manufacturing (AM), and powder metallurgy (PM), can be employed to fabricate porous Ti structures. The cost-effectiveness and efficiency of PM have resulted in its extensive use across industries for blending and compacting materials, enabling the production of implant materials with precise shapes, mechanical characteristics, and compositions [26,27]. Furthermore, the combination of space holder (SH) techniques with powder sintering allows the production of a highly porous foam structure with an accurately controlled morphology. This method, described by many authors, entails mixing and compacting space holder particles and metallic powders and then removing the SH during sintering. Magnesium (Mg) is also a viable material for space holding in the creation of porous titanium scaffolds [28,29].

Previous work demonstrated that the stiffness of porous materials improves by 13.7% and 21.1%, respectively, surpassing that of a uniform porous medium [30]. Consequently, the production of Ti foam requires high accuracy in the pore distribution [31]. While most titanium foam manufacturers offer data on porosity, mean average, and pore diameters, they often lack descriptions of mechanical properties.

Imwinkelried [32] conducted experimental research on the mechanical characteristics of open-pore Ti-foams made using the PM/SH technique. Ti-foam with porosity of 50–80% and pores ranging from 100 to 500 μm were investigated thanks to different tests: static compression, tension, bending, torsion, cyclic compression, and permeability tests. Anisotropic Ti-foam was discovered to have been utilized in the experiments. Imwinkelried [32] has deduced that the stiffness of titanium foam can be improved by controlling the porosity and making it vary from 50% (to be close to the cortical bone's porosity) to 80% for the cancellous bone.

### 1.2 Aims

In our study, we used an optimized 3D finite element model (Implant, Cortical, and Cancellous Bone) with appropriate boundary conditions in the Abaqus 6.14 software, where the mechanical properties of Ti-foam with various porosities were extracted from Imwinkelried experimental tests [32]. The objective of our study can be summarized by:

• Evaluate the precision of the mechanical properties used by modeling the stress-strain data obtained from a compression test with an artificially intelligent regression using MATLAB.
• Shown numerically the difference between solid titanium and Ti-foam used in dental implants and explain the importance of balance between energy absorption and stiffness.
• Determine numerically the most accurate and optimal porosity for titanium foam used in dental implants by modifying the porosity of titanium foam implants (50, 60, 62.5, 70, and 80%).

The novelty and originality of our work are first to implement results from the in vitro experimental work of Imwinkelried in the physical Deshpande and Fleck model of Abaqus software. Secondly, we apply it to an optimized 3D finite element model of implants and bones to investigate the optimal porosity of titanium foam implants used in dental applications and discuss the results from a biological and mechanical point of view. After the literature review and wide research on Ti foam used in dental implants, we choose this new idea to verify using FEA and the Deshpande and Fleck model the biomechanical advantages of Ti foam and the optimal porosity used in dental implants without any expensive experimental work.

## 2 Materials and methods

### 2.1 Mechanical properties

#### 2.1.1 Titanium and titanium foam

Their microstructure is the primary distinction between foam materials and solid materials. Imwinkelried experimental data on titanium foam [32] was used in our study. In this experimental test, the titanium foam was produced using a PM process with a space holder. The manufacturing process included the following operations: mixing the space holder material with the fine titanium powder grade four, pressing the resulting body, removing the space holder, and sintering. According to the British Pharmacopoeia BP E503, Imwinkelried specifies ammonium hydrogen carbonate ((NH4)HCO3) as the SH substance. The SH particles are sieved to a suitable grain size (425–710 μm). The grain size is selected to provide a final pore size (100–500 μm), which is known to prompt bone formation. In a convection oven operating at 958°C for 12 h, the space holder is removed. The components are then moved to the sintering oven where they are sintered for 3 h at 1300°C in an argon atmosphere (400 mbar). By adjusting the quantity of the SH particles, the overall porosity is changed between 50 and 80% [32]. The cell topology may describe the microstructure of metal foam, such as the relative density, size, and form of the cells [33]. The percentage of porous area in foams is denoted by the word "porosity" on a macroscopic scale. Cellular metals' mechanical behaviors are influenced by their microstructural compositions. Three regimes can be identified on the stress-strain curve for a porous material under compression: the linear elastic regime, which is related to cell bending; the plateau regime, which appears with progressive cell collapse due to elastic buckling; and the densification zone, which takes place when the cells begin to collapse throughout the material [34,35]. Imwinkelried In Vitro experimental data are widely used in Ti foam research [28,36], because of their manufacturing precision and the high reproducibility and repeatability of testing.

In our numerical study, we considered the data of Ti foam with specific porosity (50, 60, 62.5, 70, and 80%) and a low strain rate of 0.005 $s^{-1}$ determined by Imwinkelried [32] with the static compression test. The author extracted the young modulus (E) and yielded stress (Y) from stress-strain curves. The plastic Poisson's ratio ($\upsilon_{p}$) characterizing the specimen's radial shape, was established at 0.34. The compressible stress ratio (k) was found to be 0.98.

The goal of optimization is to identify the best solution to a problem within the specified parameters [37,38]. There are two popular approaches to solving optimization problems: the metaheuristic approach and the mathematical approach. The gradient-dependent mathematical techniques are reliant on the original starting point [39]. High dimensionality, non-convexity, non-linearity, and complexity are traits of real-world optimization problems [40].

**Fig. 1:**
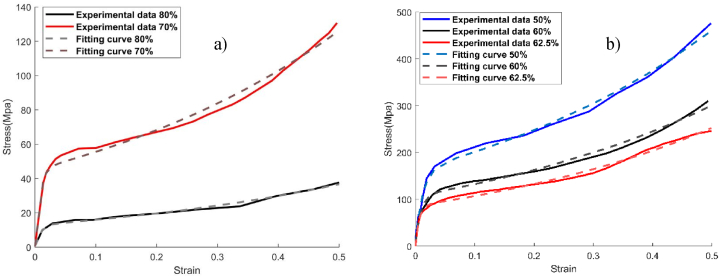
Stress-strain curve for experimental data and fitting curve with various porosities. a) 70, and 80%, b) 50, 60, and 62.5%.

Fig. 1 shows the experimental stress-strain curve with different porosities. To evaluate and describe the mechanical behavior of Ti-foam for each porosity, we use the regression learner (AI tools on MATLAB R2021b). Like polynomial, Gaussian, Fourier, and other mathematical optimizations, the exponential regression with two terms represents the best relation to describe the stress-strain variation of Ti foam [41]. It can be defined by the following equation (1):

(1) $$\sigma \left(\varepsilon \right)=ae^{b*\varepsilon }+ce^{d*\varepsilon }$$

where σ represents the compression stress (MPa) and ε the corresponding strain(mm/mm). Table 1 gives the computed constants *a*, *b*, *c*, *d* and *R-square* for each porosity.

**Table 1:** The constants *a*, *b*, *c*, and *d* for exponential model with the corresponding *R-square*.

Porosity (%)	a	b	c	d	R-square
**50**	164 ± 9	2.07 ± 0.2	−158.3 ± 11	−82.54 ± 15	0.996
**60**	108.2 ± 4	2.04 ± 0.1	−102.3 ± 9	−111.8 ± 20	0.993
**62.5**	86.61 ± 1	2.14 ± 0.2	−86.82 ± 8	−163.4 ± 22	0.993
**70**	45.27 ± 2	2.051 ± 0.1	−44.8 ± 7	−132.7 ± 33	0.990
**80**	12.98 ± 1	2.08 ± 0.2	−12.4 ± 3	−114.3 ± 35	0.992

The exponential fitting generates a high precision relation where the *R-square* is higher than 0.99. Then all porosities of titanium foam represent the same mathematical variations between compressive stress and strain. The difference between Imwinkelried data and the correlated curve is represented in Fig. 1a) for porosity 70 and 80%, and Fig. 1b) for others 50, 60, and 62.5%.

Young's modulus may be calculated using a linear fitting on the elastic zone of the stress-strain variation curve. The yield stress is the stress at which residual deformation reaches 0.2% of the elastic limit. These mechanical properties of each porosity are filled out in Table 2, and they will be used in the Deshpande and Fleck model in Abaqus software later.

**Table 2:** The Young modulus and yield stress of Ti foam with various porosities.

Porosity (%)	Young modulus E(GPa)	*Yield stress*$\sigma_{y}$*(MPa)*
**50**	40.47	170.63
**60**	16.32	105.15
**62.5**	13.82	52.362
**70**	4.32	25.476
**80**	0.711	15.3

The energy absorbed per unit volume of the material is related to the surface under the stress-strain curve. Additionally, the energy absorption is influenced by the cell morphology, base material, foam density, and other factors affecting the length of the visible plateau in the compression curve of these materials [42]. The energy absorption per unit volume (W) can be described by the equation (2):

(2) $$W=\int_{0}^{\varepsilon_{m}}\sigma \left(\varepsilon \right)\;d\varepsilon$$

The energy absorption for each porosity was calculated from the stress-strain data exposed in Fig. 1 using MATLAB. To further emphasize the accuracy of the mathematical exponential equation, we compared the energy under experimental and predicted stress-strain curves using the equation (2) and a little difference exists for all porosities (Table 3).

**Table 3:** Energy absorption (Mj/ $m^{3})$ for experimental data and fitting curve with different porosities.

*Porosity (%)*	*Energy Absorption (Mj/*$m^{3}$*)*	
	*Experimental data*	*Fitting curve*
**50**	141.129	140.991
**60**	90.462	91.451
**62.5**	77.038	76.778
**70**	38.360	38.648
**80**	11.213	11.296

According to Ref. [32], the stiffness of the solid titanium grade 4 is: Young's modulus E = 110 GPa, Poisson's ratio ν = 0.33, and the yield strength $\sigma_{y}$ = 650 MPa.

#### 2.1.2 Cortical and cancellous bone

Cortical and cancellous bones are the two main categories of bones. Compact bones called cortical bones are in charge of mobility, structural stiffness, and mechanical strength. They represent 80% of the mass of the bones. Cancellous bones are soft, spongy bones that give the cortical bones structural support, flexibility, and weight loss. In terms of porous structure, cortical bone has a porosity of less than 10% while cancellous bone has porosities ranging from 50% to 90% [43].

To simplify our numerical simulation, cortical and cancellous bones are assumed to be isotropic, homogenous, and linearly elastic. This assumption is used due to our simulation goal to show the first chewing function during 0.5 s, where the stress does not exceed the elastic zone. In real cases, this assumption is not useful to simulate the fatigue of the implant versus time. Those human mechanical properties are influenced by their mandibular and gum health, genetic makeup, the pace of healing, and a few other variables. As a result, the information in Table 4 refers to a person with normal tissues [44].

**Table 4:** Mechanical properties of bones in the current model [44].

	Young's modulus (GPa)	Poisson's ratio
***Cortical bone***	14.5	0.323
***Cancellous bone***	1.37	0.3

### 2.2 3D optimized model

The Finite Element Method (FEM) numerically discretizes a continuous structure into simplified small elements, in order to model complex mechanical and physical phenomena for engineering applications. It is now a vital tool in several technical domains, particularly in dental implantology and biomechanics. Here are some reasons why the FEM is important in the dental implant field: Biomechanical Analysis, Optimization of Implant Design, Predicting Failure Modes, Stress Analysis, and Personalized Treatment Planning [45].

**Fig. 2:**
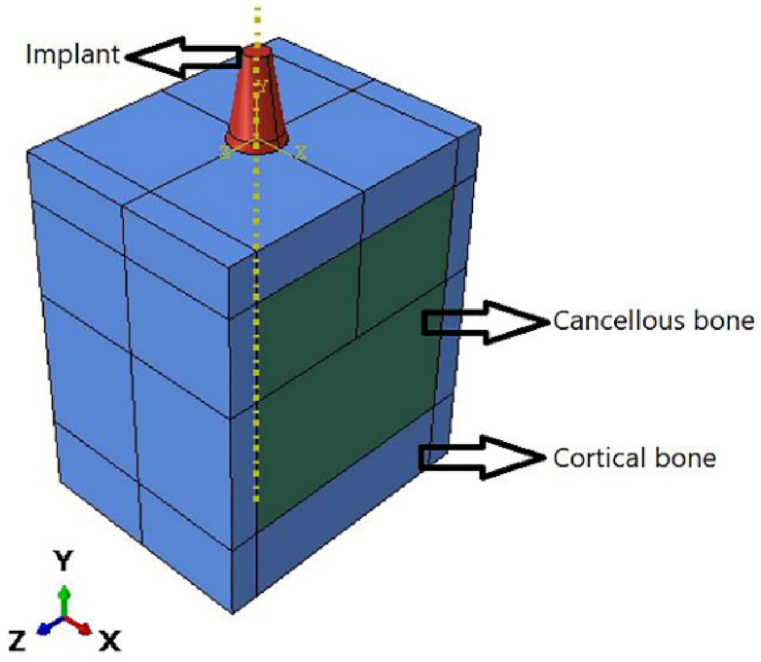
3D optimized finite element model.

**Fig. 3:**
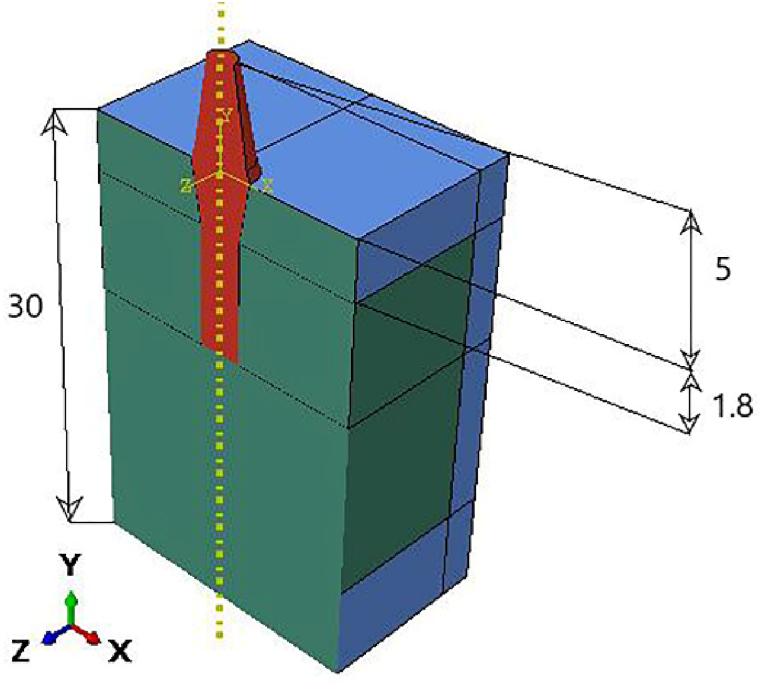
Dimensions (mm) of the 3D model.

To initialize our simulations, a 3D finite element model was generated using Abaqus 6.14. Cortical and cancellous bone with specific implant dimensions was modeled with an optimized shape Fig. 2. The highest level of stress is applied to the top section of the bone. Suggests that the bone receives a direct signal from the imposed load to the crown [46]. For this reason, to improve our mesh quality and find the stress distribution from implant to bones with high precision at the neck region, we optimized our implant by suppressing the corresponding thread and replacing that with tie constraints between implants and bones. The specific tie constraint can be applied to simulate how the implant is anchored or connected to surrounding structures. This constraint can help to accurately model the implant's behavior and optimize its thread design accordingly. The other contact between parts is considered frictional with a coefficient of 0.5 [46]. The implant diameter is 4.1 mm, and the thickness of the cortical bone varies within 1.3–2.0 mm according to Ref. [47] shown in Fig. 3.

**Fig. 4:**
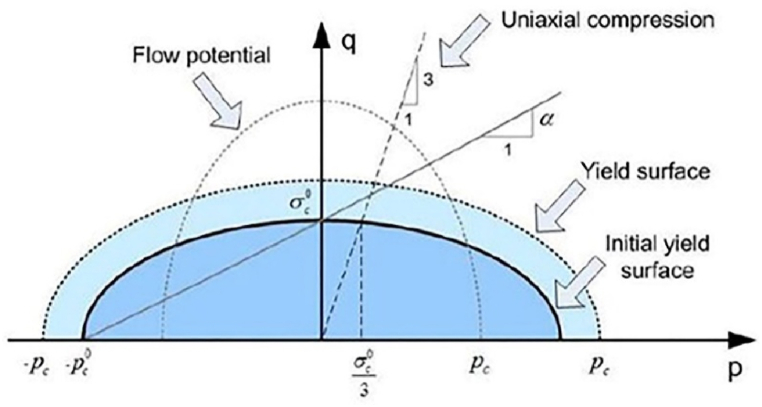
Yield surfaces and flow potential of crushable foam model [49].

The Deshpande and Fleck model of crushable foam included in Abaqus software is used in our 3D finite element simulation [48]. The simulation utilizes a shape factor parameter to approximate the effect of mean stress on the yield function of foam. This shape factor is used to distinguish the plastic behavior of foam metals, and it creates an elliptical stress aspect ratio. The yield function (*Y*) is represented by equation (3) and presented in Fig. 4 [48,49].

(3) $$Y=\sqrt{q^{2}+p^{2}\alpha^{2}}-B$$

where p is the mean stress and q represents the Von Mises equivalent stress. B defines the size of the ellipse and α the shape factor of the surface.

This specific model is used in our analysis to simulate the model's response to the loads and boundary conditions defined in subsequent parts.

### 2.3 Finite element model

#### 2.3.1 Mesh

**Fig. 5:**
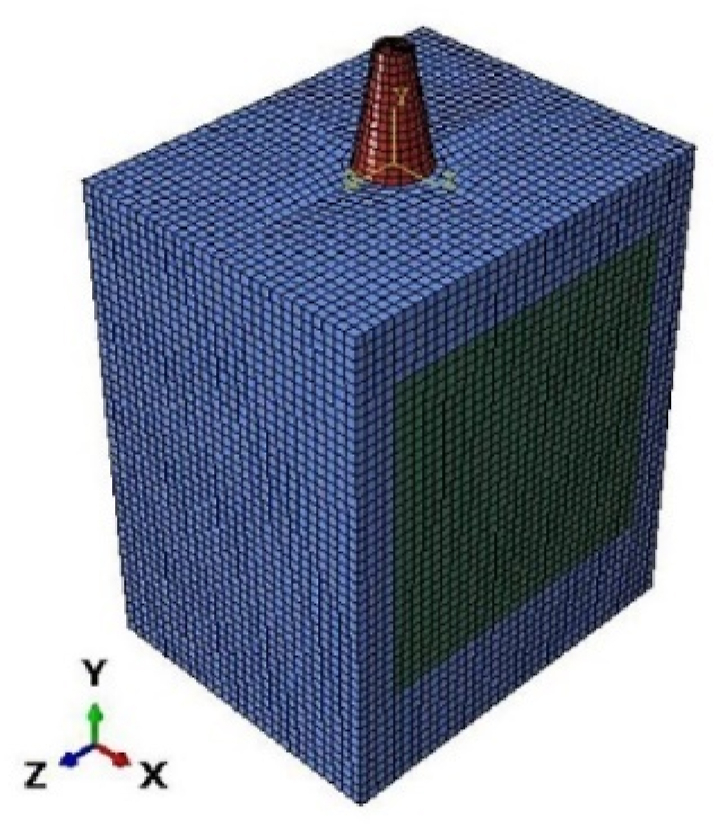
Meshed model.

In this section, we describe the mesh process of implants, cortical bone, and cancellous bone for the 3D model shown in Fig. 5. The model is divided into parts and meshed with a global size of 0.3 mm for the implant and 0.7 mm for the bone using quadratic hexahedron elements (C3D8RH). The quadratic hexahedral elements offer distinct advantages over other mesh forms, such as tetrahedron or pyramid elements: higher accuracy in representing complex geometries, volume meshing efficiency, reduced element count, reduced mesh sensitivity, and compatibility with CAD models.

The resulting mesh has 48026 elements which are of particularly high quality without any poor elements. The mesh quality is assessed and optimized through specific parameters like geometric deviation factor, average aspect ratio, minimum edge length, and maximum angle of quadrilateral faces. These parameters are summarized in Table 5.

**Table 5:** Meshing parameters and quality of the model.

	*Number of elements*	*Avg Aspect ratio*	*Avg deviation factor*	*Avg Max angle*	*Avg Min edge length*
*Bone*	46316	1.14	8.01 $*10^{-4}$	94	6.55 $*10^{-4}$
*Implant*	1710	1.75	0.0367	107	3.3 $*10^{-4}$

**Fig. 6:**
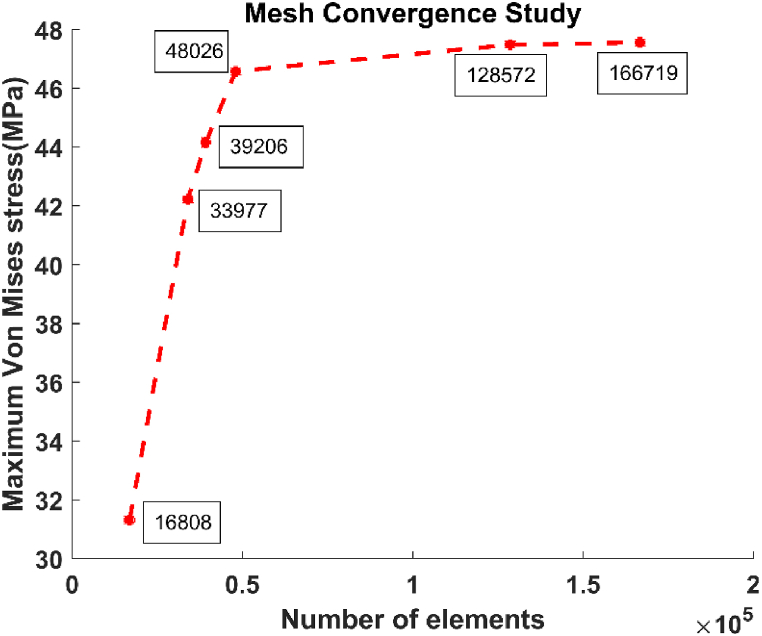
Convergence study: influence of the number of elements on the maximum stress.

Since the FEA only provides an approximate solution, a convergence analysis of mesh is conducted to ensure that the mesh quality doesn't distort the provided results. The mesh convergence study shows that doubling the mesh resolution in peak stress-prone areas has less than 2% impact on results. We expose this slight difference by reporting the maximum von Mises stress (MPa) of the whole model concerning the number of elements in the case of a titanium foam implant with 80% porosity (Fig. 6). This porosity was chosen because it's the most affected by the refinement of elements due to the high deformations obtained for this high porosity 80% seen in the results section.

#### 2.3.2 Boundary conditions

**Fig. 7:**
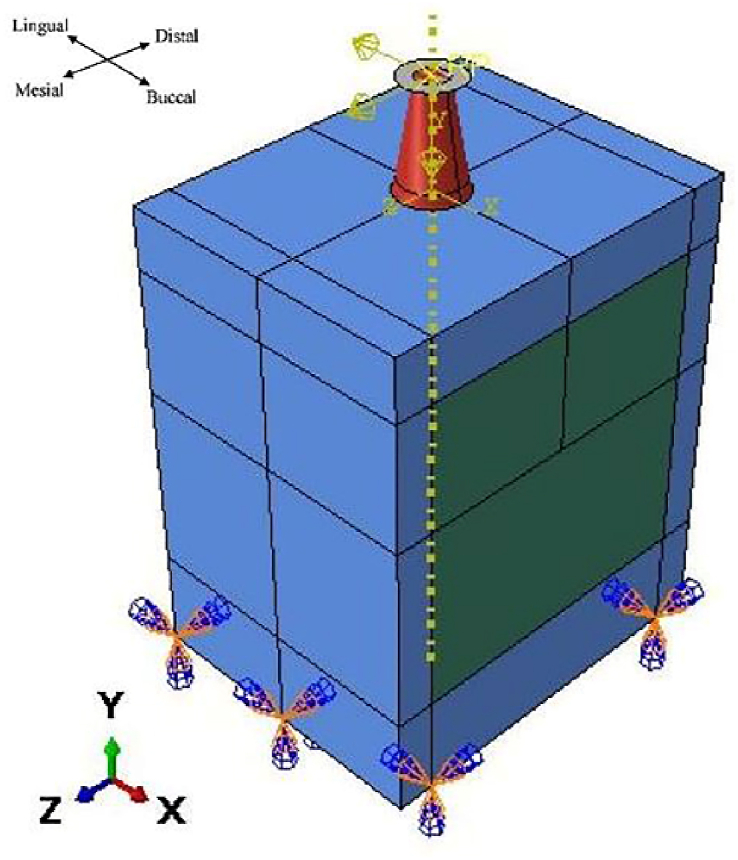
Load and boundary conditions.

**Fig. 8:**
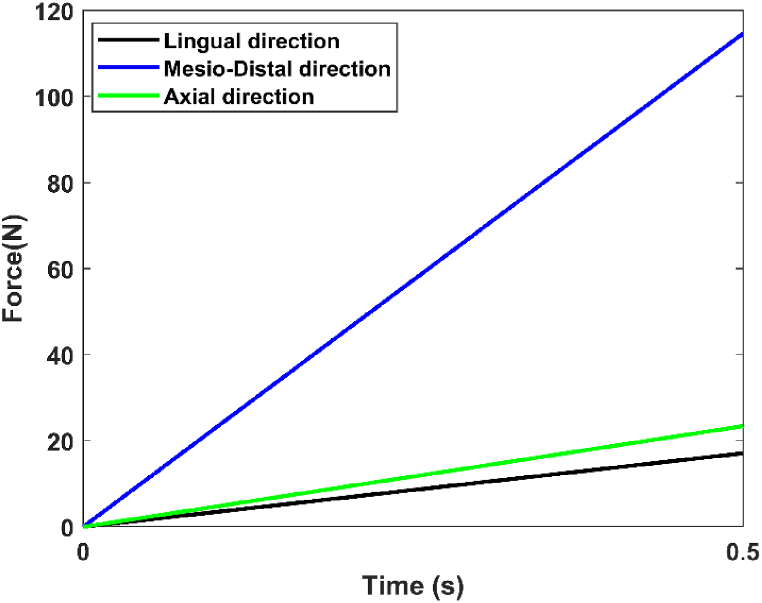
Dynamic load in 0.5 s [19].

The lower surfaces of the cortical bone are considered fixed supports (zero DOF, (U_i_ = UR_i_ = 0; i = 1,2,3)), and the dynamic load simulates average chewing forces of 17.1 N in the lingual direction (-X), 114.6 N in the axial direction (-Y), and 23.4 N in the mesiodistal direction (+Z) (Fig. 7) [19]. These forces are combined to produce a masticatory force of 118.2 N at a roughly 75°angle to the occlusal plane. The force magnitudes and acting points were selected based on Mericske-Stern's clinical work. The simulation is an explicit dynamic simulation with a period of 0.5 s, representing the first chewing phase based on Kayabasi et al.(Fig. 8) [19]. To take into account the worst case, the force applied during chewing is the one used to model the strongest loads in dental applications. It's crucial to underline that each person's chewing force differs depending on variables like biting strength, dental alignment, and jaw function.

## 3 Results

To find the distribution of stress and strain in dynamic dental applications, five criteria can be used: Von Mises (VM), Tresca, maximum principal (S1), minimum principal (S3), and hydrostatic pressure. Since the latest three presented a variety of unusual biomechanical stress, only Tresca and VM criteria displayed biomechanically appropriate stress [50]. The fact that foam materials can undergo plastic deformation in the plateau regime justifies the use of VM stress, which is more accurate and appropriate for our study.

### 3.1 Implants

**Fig. 9:**
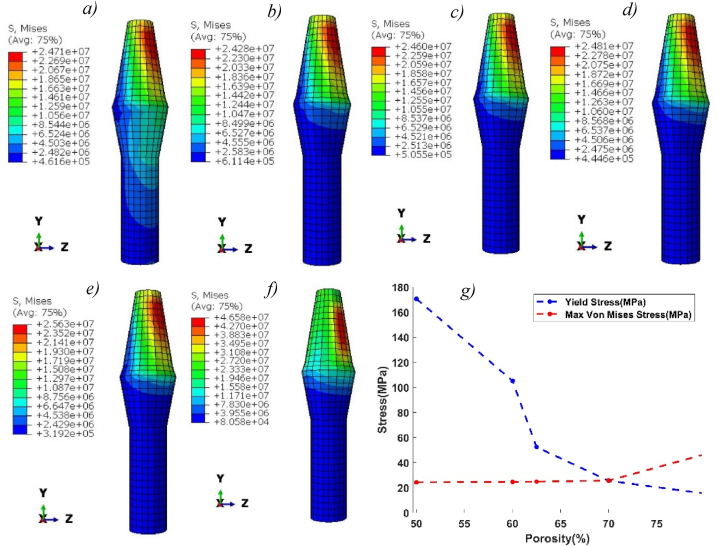
Von Mises stress (Pa) distribution in titanium and Ti-foam implant with different porosities; a) Pure titanium b) P = 50%, c) P = 60%, d) P = 62.50%, e) P = 70%, f) P = 80%; (g) Comparison between Max von Mises stress and Yield stress for each porosity.

Fig. 9 (*a-f*), shows the Von Mises stress distribution along implants for pure titanium and Ti-foam with different porosities. The stress distributions are inhomogeneous and depend on the direction of masticatory forces. The maximum concentration of stress occurs near the neck region. In the case of pure titanium, the stress is distributed along the length of the implant more evenly than in the case of Ti-foam with various porosities, where the stress is negligible after the neck region. Maximum stress increases with porosity, rising from 24.2 MPa with 50% porosity to 46.5 MPa with 80% porosity (Table 6). We observe that the stress level between pure titanium and Ti-foam with 62.5% porosity is approximately the same. In Fig. 9 (*g*), the comparison between the yield stress and the maximum stress on the implant shows that where the porosity is higher than 70%, the maximum stress exceeds the yield stress value. So, the risk of implant fracture occurs.

**Table 6:** The maximum value of Von Mises stresses (MPa) for each part of the model with different porosities.

			Porosity (%)				
		Titanium	50	60	62.5	70	80
***Von Mises stress (MPa)***	*Implant*	24.71	24.28	24.6	24.81	25.63	46.58
	*Cortical bone*	5.427	8.236	8.357	8.463	9.619	21.53
	*Cancellous bone*	0.53	0.45	0.476	0.498	0.592	1.16

**Fig. 10:**
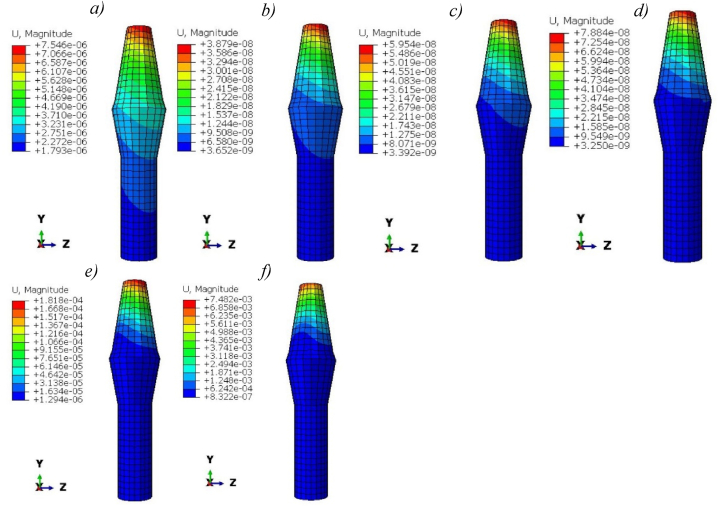
Magnitude of deformation (m) for titanium and Ti-foam implant with different porosities; a) Pure titanium b) P = 50%, c) P = 60%, d) P = 62.50%, e) P = 70%, f) P = 80%.

Fig. 10 shows the magnitude of deformation of titanium and Ti-foam implants with different porosities. Maximum deformations in pure titanium implants are greater than Ti-foam with 50, 60, and 62.5% porosity, but less than those with porosities of 70 and 80%. We notice that, in cases of 50, 60, and 80% porosities, the deformation remains negligible away from the neck region in the -Y direction. The magnitude of deformations increases with porosity.

### 3.2 Cortical bone

**Fig. 11:**
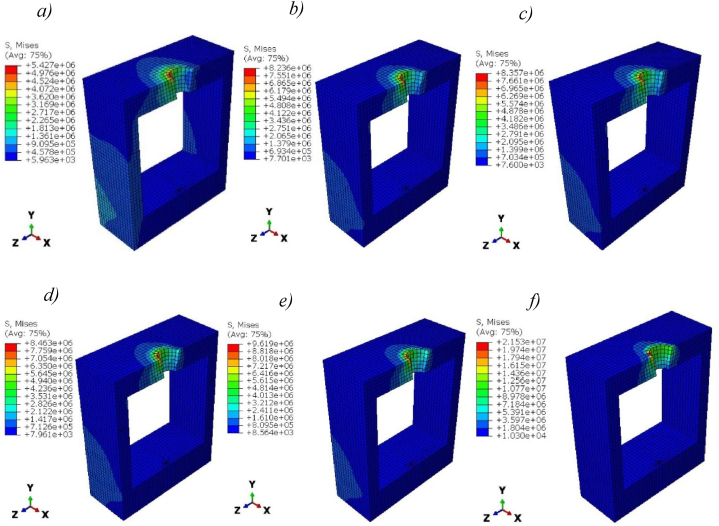
Von Mises stress (Pa) distribution in Cortical bone for titanium and Ti-foam implants with different porosities; a) Pure titanium b) P = 50%, c) P = 60%, d) P = 62.50%, e) P = 70%, f) P = 80%.

Fig. 11 presents the Von Mises stress distribution within the surrounding cortical bones in the case of solid titanium and Ti-foam implants. Stresses in titanium foam implants with different porosities are higher than those in solid titanium, especially at the neck region where the maximum value occurs. That means more loads can be transferred to the cortical bone in the case of foam implants. The maximum stress at the neck region of cortical bone increases with porosity from 8.2 MPa for 50% porosity to 21.5 MPa for 80% (Table 6). In another region of surrounding cortical bone away from the neck interaction surface, a little difference in stress was shown.

The distribution of stress was higher in the cervical line at the bone-implant interaction surface for all porosities; this area has experienced higher levels of stress.

The porosity of Ti foam affects the stress distribution in the cortical bone due to the differentiation in the mechanical properties of the implants. In the case of 80% porosity, the distribution of stress is higher than others due to their lower young modulus (0.71 GPa).

**Fig. 12:**
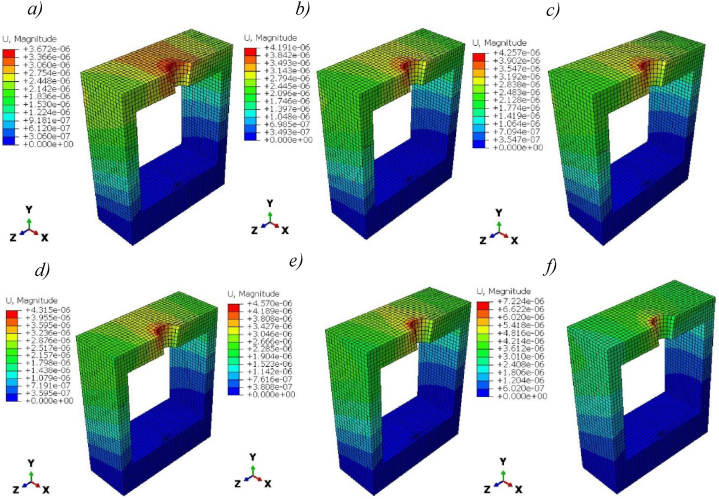
Magnitude of deformation (m) in Cortical bone for titanium and titanium foam implants with different porosities; a) Pure titanium b) P = 50%, c) P = 60%, d) P = 62.50%, e) P = 70%, f) P = 80%.

The magnitude of deformation of cortical bones presents a little difference between solid titanium and Ti-foam with different porosities, as shown in Fig. 12.

### 3.3 Cancellous bone

**Fig. 13:**
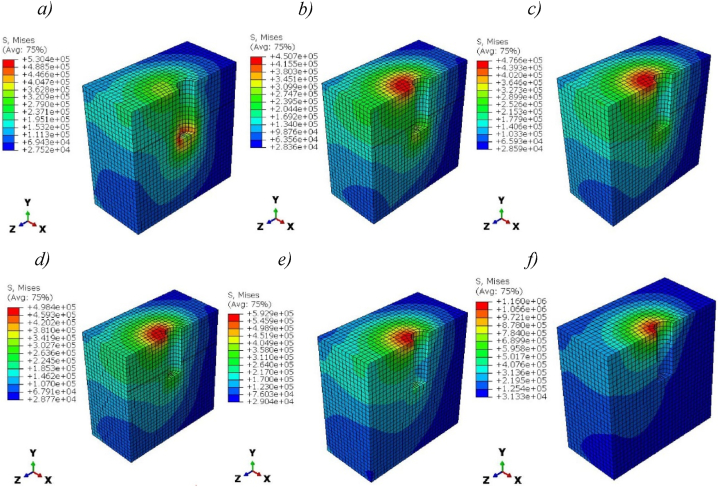
Von Mises stress distribution (Pa) in Cancellous bone for titanium and titanium foam implants with different porosities; a) Pure titanium b) P = 50%, c) P = 60%, d) P = 62.50%, e) P = 70%, f) P = 80%.

Fig. 13 shows the Von Mises stress distribution within the surrounding cancellous bones in the case of solid and foam titanium implants. The maximum stress for pure titanium implants occurs at the bottom face contact between implants and cancellous bone, with a small value of 0.53 MPa. In the case of Ti-foam implants, the maximum stress occurs at the top contact face between cortical and cancellous bone, ranging from 0.45 MPa for 50% porosity to 1.16 MPa for 80% (Table 6). In other regions, we show a little difference, and we notice that the stress transferred to the cancellous bone is very small.

**Fig. 14:**
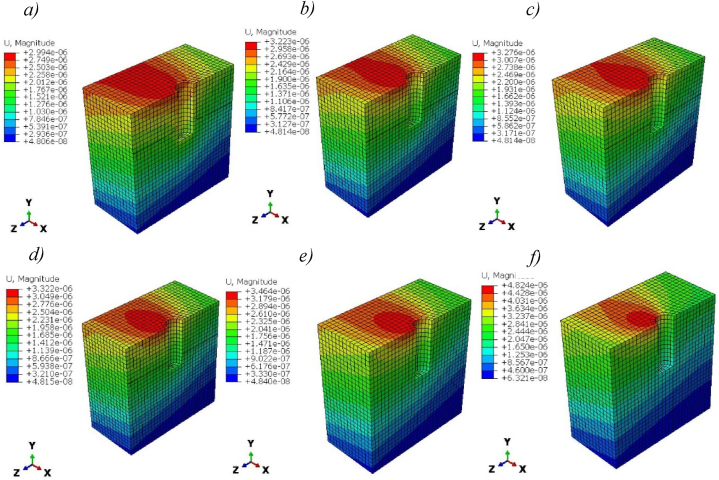
Magnitude of deformation (m) in Cancellous bone for titanium and Ti-foam implants with different porosities; a) Pure titanium b) P = 50%, c) P = 60%, d) P = 62.50%, e) P = 70%, f) P = 80%.

Due to the low transferred stress, the magnitude of deformation of cancellous bones presents a small value with little difference between solid and foam titanium implants with different porosities, as shown in Fig. 14.

## 4 Discussion

The solid titanium is stiffer than the titanium foam, for this reason, the Ti-foam implants transferred more loads to the surrounding bones (Fig. 11). So, it's more beneficial to use titanium foam to reduce the probability of a stress shielding problem. This can cause the bone to weaken and deteriorate over time due to the lack of mechanical stimulation [21,51]. Furthermore, solid titanium has a higher Young's modulus than mineralized tissues. As a result, it causes insufficient loading of the underlying bone tissue and stress shielding, which eventually leads to implant loosening, bone resorption, and failure.

Then, to choose the accurate porosity for Ti-foam dental implants, a balance should be found between stiffness and energy absorption. Titanium foam implants with porosities between 70 and 80 % present a high magnitude of deformation compared to lower foam porosities, and this can affect the implant connections with surrounding bones because of their low stiffness (Fig. 10). So clinically the use of Ti foam dental implants with porosity higher than 70% can cause a fracture of implants under natural masticatory loadings, or disconnection with the surrounding bones due to their low stiffness and energy absorption (Table 3). A simulation depicting the actual fracture of an implant is observable within a fatigue-versus-time Finite Element Model (FEM). While the use of porosities 50, 60, and 62.5% is more interesting because of the smaller gap in stress distributions.

We show that the stress leveling of Ti-foam with a porosity of 62.5% and solid titanium implants are approximately the same, with an equal maximum value and location of von Mises stress (Fig. 9). This similarity is due to the rapprochement of Young's modulus between the cortical bone (14.5 GPa) and Ti-foam with this specific porosity (14.8 GPa) according to Table 2. For this reason, the Ti-foam implants with a porosity of 62.5% are a great solution among the tested porosities to be used for dental applications. In clinical practice, the young's modulus of the employed implant is close to 14.5 GPa, which corresponds to the stiffness of cortical bone, in order to lessen stress shielding problems without sacrificing implant stiffness and lifetime.

Because of the high stiffness of cortical bone compared to cancellous bone, the stress transferred to the cancellous surrounding bone is very low [52].

In the FEM with a dynamic explicit problem, we used a hexahedron structure mesh with an appropriate element quality applied to an optimized shape of dental osseointegration. We obtained very interesting and promising results based on these numerical improvements, with an accuracy of less than 2% after the convergence study. However, we have some limitations, like that the bone properties are considered to be isotropic and homogeneous, but the biological tissue is anisotropic and porous.

Surface chemistry influences protein, bacteria, and cell adhesion to implants. Dental implant companies use hydrophilic surfaces, promoting better osseointegration as cells move differently on hydrophilic surfaces [53].

Even though solid titanium implants present some disadvantages in osseointegration the surface treatment can improve their biomechanical ability. Surface treatment is crucial for improving titanium mechanical properties (*Anodic Oxidation, Chemical Vapor Deposition (CVD)),* bioactivity, and osseointegration (*Sandblasting/Grit-Blasting, Acid-Etching, Alkaline Treatment, Acid Treatment),* antibacterial effect *(Antibiotic and Nonantibiotic Organic Coatings - Inorganic Antimicrobial Coatings)* [[1], [2], [3],^54^].

From a biological point of view, the porous titanium foam facilitates vascularization and bone regeneration by drawing and allowing cells from the neighboring bone tissue to penetrate its interior [2,26]. To alleviate the issue of stress concentrations within the pores, one can employ silanization of the pore walls, followed by filling them with poly-methylmethacrylate (PMMA) [54].

High manufacturing precision is necessary to achieve the exact porosity required for titanium foam dental implants. Powder metallurgy provides several advantages when it's combined with the SH. Powder metallurgy techniques are simpler to industrialize, less expensive, and less consuming time in comparison to rapid prototyping methods such as SLM or 3D printing [55]. For titanium, its strong chemical reactivity with surrounding mold materials and gases, and high melting point make solid foaming using powder metallurgy more appealing than liquid foaming methods. Additionally, the pores are randomly distributed and exhibit a wide range of diameters [56]. This could be seen as a drawback compared to the SLM method, which provides more precise control over the distribution of pores. Albert Barba et al. [57] demonstrated that only foams with spherical macro-pores significantly promoted ectopic bone growth, contrary to those with prismatic macropores created by 3D printing. This unique property makes the PM/SH combination an extremely attractive approach for manufacturing porous metal for osteoregeneration.

Then, PM/SH is an appropriate approach to reduce these homogeneity problems of Ti-foam. The ammonium bicarbonate used in the Imwinkelried experiment is the best SH because it can be completely and easily removed. Its moderate decomposition temperature ensures minimal uptake of impurities like carbon, nitrogen, and oxygen [58]. The mechanical properties of the specimen are directly influenced by its manufacturing imperfections. This is due to the uneven geometry of the pores and pre-existing crack tips. Some studies have shown that samples with irregular pores experience yielding at lower stress compared to samples with regularly shaped pores [36].

Previous experimental and numerical works only show an acceptable interval of porosity used in dental implants [7,[20], [21], [22], [23]], but the novelty of our study is to indicate the accurate porosity of Ti foam at 62.5% using an optimized FEM with the intermediary Deshpande and Fleck model.

## 5 Conclusions and future works

In the current study, we extracted the mechanical properties of Ti-foam used in dental implants from published data with different porosities (50, 60, 62.5, and 80%). We describe the stress-strain response with an artificially intelligent exponential fitting. A good agreement is obtained with high porosity, especially between the elastic and plastic zones. Then, in Abaqus software, we generate a 3D explicit finite element model with implants, cortical, and cancellous bone using crushable foam sections. Our goal was to numerically determine the more adequate porosities used in dental implants after applying the corresponding masticatory forces.

The numerical results show that the titanium foam transferred more loads than solid titanium to the surrounding bones due to its low stiffness and high energy absorption. Then the porous foam reduces the stress shielding problems. The 62.5% porosity of Ti-foam is more interesting to be used than other tested porosities because of the similarity of stress leveling with solid titanium implants.

Based on these findings, our work demonstrates that Ti foam dental implants can significantly improve implant success rates and patient outcomes. It can guide in developing calculation methodology to enable manufacturers and surgeons to select the efficient implant with the best porosity depending on bone quality and stiffness range, leading to better clinical outcomes and patient satisfaction.

In the future, new precise methods of manufacturing, such as 3D printing, can reduce the production time and cost of this type of implant without sacrificing accuracy or mechanical characteristics.

In our future works, we will enhance these dynamic numerical simulations (full and partial osseointegration) by incorporating the actual geometry of the titanium foam implant with various porosity levels, and the inhomogeneous structure of bone with anisotropic properties. In addition, we will develop a new AI mathematical relation between mechanical properties and fatigue behavior of implants. This will enable a comprehensive analysis of the fatigue behavior of thread connections under varying load configurations.

## Ethics approval

The submitted manuscript is original and has not been published elsewhere in any form or language.

## Data availability

All data are available on request to the corresponding author.

## Funding

This research received no external funding.

## CRediT authorship contribution statement

**Hussein Farroukh:** Writing – review & editing, Writing – original draft, Visualization, Validation, Software, Methodology, Investigation, Formal analysis, Data curation, Conceptualization. **Fouad Kaddah:** Writing – review & editing, Writing – original draft, Validation, Supervision, Project administration, Formal analysis, Conceptualization. **Toufic Wehbe:** Writing – review & editing, Writing – original draft, Visualization, Validation, Supervision, Methodology, Formal analysis, Conceptualization.

## Declaration of competing interest

The authors declare that they have no known competing financial interests or personal relationships that could have appeared to influence the work reported in this paper.
